# A Clinically Applicable Approach to the Classification of B-Cell Non-Hodgkin Lymphomas with Flow Cytometry and Machine Learning

**DOI:** 10.3390/cancers12061684

**Published:** 2020-06-24

**Authors:** Valentina Gaidano, Valerio Tenace, Nathalie Santoro, Silvia Varvello, Alessandro Cignetti, Giuseppina Prato, Giuseppe Saglio, Giovanni De Rosa, Massimo Geuna

**Affiliations:** 1 Department of Clinical and Biological Sciences, University of Turin, 10043 Orbassano, Italy; valentina.gaidano@unito.it (V.G.); giuseppe.saglio@unito.it (G.S.); 2 Division of Hematology, A.O. SS Antonio e Biagio e Cesare Arrigo, 15121 Alessandria, Italy; 3 Department of Electrical and Computer Engineering, University of Utah, Salt Lake City, UT 84112, USA; 4 Laboratory of Immunopathology, Division of Pathology, A.O. Ordine Mauriziano, 10128 Turin, Italy; nsantoro@mauriziano.it (N.S.); gderosa@mauriziano.it (G.D.R.); 5 University Division of Hematology and Cell Therapy, A.O. Ordine Mauriziano, 10128 Turin, Italy; silvia.varvello@unito.it (S.V.); alessandro.cignetti@unito.it (A.C.); 6 Division of Pathology, San Lazzaro Hospital, ASL CN2, 12051 Alba, Italy; giusi.prato70@gmail.com

**Keywords:** lymphoma, non-hodgkin, classification, artificial intelligence, machine learning, flow cytometry

## Abstract

The immunophenotype is a key element to classify B-cell Non-Hodgkin Lymphomas (B-NHL); while it is routinely obtained through immunohistochemistry, the use of flow cytometry (FC) could bear several advantages. However, few FC laboratories can rely on a long-standing practical experience, and the literature in support is still limited; as a result, the use of FC is generally restricted to the analysis of lymphomas with bone marrow or peripheral blood involvement. In this work, we applied machine learning to our database of 1465 B-NHL samples from different sources, building four artificial predictive systems which could classify B-NHL in up to nine of the most common clinico-pathological entities. Our best model shows an overall accuracy of 92.68%, a mean sensitivity of 88.54% and a mean specificity of 98.77%. Beyond the clinical applicability, our models demonstrate (i) the strong discriminatory power of MIB1 and Bcl2, whose integration in the predictive model significantly increased the performance of the algorithm; (ii) the potential usefulness of some non-canonical markers in categorizing B-NHL; and (iii) that FC markers should not be described as strictly positive or negative according to fixed thresholds, but they rather correlate with different B-NHL depending on their level of expression.

## 1. Introduction

Lymphoid neoplasms are a very heterogeneous group of malignancies, ranging from indolent lymphomas to extremely aggressive entities. Different lymphomas require different therapies, show different responses to treatments and are generally associated with very different prognosis. For these reasons, the WHO classification [1,2] of lymphoid neoplasms is of huge importance in routine clinical practice, and it is based on morphology, immunophenotype, genetic abnormalities, and clinical features.

The immunophenotype can be obtained through flow cytometry (FC), immunohistochemistry (IHC), or ideally both, as each bears advantages and disadvantages. While the architecture of tissues is preserved in IHC, FC allows to (i) evaluate millions of cells simultaneously, (ii) easily define a clonal population, (iii) evaluate each single cell for different markers, and (iv) specifically gate the clonal population and follow it in different panels. Moreover, FC can be extensively utilized in fine needle aspiration (FNA) samples, being more sensitive and specific than cytology alone [3,4] and on peripheral blood (PB) samples of lymphomas undergoing leukemization. It is also worth to be noted that FC can give information about intracellular antigens, such as Bcl2, Bcl6, and Ki-67, at the very same way as IHC does, if the staining procedure is performed correctly. In general, FC provides faster and less biased results compared to IHC, since data are expressed in a quantitative way; in fact, even though the gating strategy may introduce some subjectivity in FC, when gates are designed to identify a specific population (defined by the immunological markers) the subjectivity is limited.

Many of these features have already been stated long ago [5,6], but despite all these advantages, FC is frequently restricted to the diagnosis of Chronic Lymphocytic Leukemia (CLL) and non-Hodgkin lymphomas (NHL) with a PB or bone marrow (BM) involvement. Only few laboratories routinely use FC for tissue biopsies, and even fewer perform a wide characterization with extended panels of monoclonal antibodies. The reasons for this underuse could lie both at the practical and at the theoretical level: difficulties in sample preparations, the belief that morphology and IHC are generally enough and, above all, the lack of a long-standing practice and of a vast literature in support.

As an answer to these problems, we applied machine learning (ML) algorithms to a large dataset of B-NHL immunophenotype traces, in order to generate a solid and clinically applicable predictive system, leveraging the vast experience accumulated by our Laboratory in the last 16 years.

Broadly speaking, ML allows computers to learn from their own experience. In other words, ML algorithms are able to analyze large amounts of existing data with the aim of deducting rules or recurrent patterns that are eventually utilized to solve similar future problems. Similar to human beings, ML algorithms encompass a period of “training”, in which they learn a predictive model, and a period of “validation”, where the model is applied to never-before-seen cases. In the medical field, several tools have already been implemented: notable examples are the interpretation of radiological [7,8], histopathological [9,10], and fundus oculi images [11,12], as well as the prediction of clinical outcomes based on electronic health records [13]. As FC produces a large amount of data, whose interpretation needs high analytical skills and a conspicuous experience, it naturally represents the perfect field of application for ML algorithms: accordingly, ML tools have already been applied to different FC steps, from data preprocessing [14,15] to disease or minimal residual disease (MRD) detection [16,17]. In the B-NHL field, previous papers showed that automated tools could distinguish between different types of lymphomas in a one-to-one comparison (e.g., small cell lymphoma vs. mantle cell lymphoma) with a good to optimal accuracy [18,19]. More recent works have started to apply ML tools to raw FC data, to comprehensively analyze and classify blood samples with suspected B-cell lymphomas [20,21,22] leveraging a no-human-in-the-loop approach. In this work, on the contrary, we wanted to design an easy-to-interpret, decision-support technique, which could be manually inspected by FC operators and that could help them classify future samples from every source (blood and non-blood samples). Beyond the clinical applicability, an interpretable tool could suggest possible improvements to the current laboratory standards, such as the selection of markers that guarantee the best diagnostic accuracy. In this regard, we decided to apply classification trees, which are characterized by a simple and interpretable structure, to our database of 1465 B-NHL samples, extensively characterized in FC and histologically/cytogenetically labelled according to the WHO classification; we obtained four predictive systems that could categorize B-NHL in up to nine of the most common clinico-pathological entities with optimal accuracies.

## 2. Results

### 2.1. Database and Models Design

At the end of data preprocessing (refer to Section 4.3), we obtained a conspicuous database containing 1465 immunophenotype traces from as many samples collected in the 2003–2019 period. Samples derive from different sources (PB, BM aspirates, lymph node and tissue biopsies or FNA, liquor, bronchoalveolar lavage, pleural and peritoneal effusions) of patients affected by the most common mature B-NHL, and in particular: Chronic Lymphocytic Leukemia (CLL), Diffuse Large B-cell Lymphoma (DLBCL), Burkitt Lymphoma (BL), Follicular Cell Lymphoma (FCL), Hairy Cell Leukemia (HCL), Splenic Lymphoma (SL), Mantle Cell Lymphoma (MCL), Marginal Zone Lymphoma (MZL), Lymphoplasmacytic Lymphoma (LPL). Please refer to Section 4.2 and Section 4.3 for a detailed description of data collection and preprocessing.

Each sample is characterized with an extended immunophenotype; however, these samples were studied as part of the routine clinical practice, which also changed during the 16 years of samples collection. The result is that not every sample is characterized for all markers. This aspect is particularly important for MIB1 and Bcl2, as these two intracellular markers are mostly utilized on samples other than PB or BM aspirates, i.e., lymph nodes, tissue biopsies, and effusions.

First of all, we decided to eliminate surface markers with a high missing-value rate (refer to Section 4.1); for the remaining markers, missing values were filled-in with class-wise mean values, as described in Section 4.3. Moreover, considering the peculiarity of intracellular markers, we decided to generate multiple predictive models, with and without MIB1 and Bcl2; this allowed us to overcome the inevitable problem of missing values in retrospective studies, obtaining the most significant results while avoiding artifacts.

We started by applying ML techniques to the entire training set, removing MIB1 and Bcl2 (Model I). Secondly, we introduced MIB1 and Bcl2, but we removed HCL and SL from the analyses, as samples from these two subclasses had not been consistently evaluated for MIB1 and Bcl2 (Model II).

Thirdly, in order to analyze the net benefit induced by MIB1 and Bcl2, we analyzed the very same dataset used to generate Model II excluding MIB1 and Bcl2, thus obtaining Model III. Finally, in order to demonstrate that the importance of MIB1 and Bcl2 was not an artifact due to the filling methodology utilized, we analyzed non-blood samples only, which are for the most part characterized with these two markers (74.81% of the samples for Bcl2, 96.35% for MIB1); in this model, named Model IV, we had to remove HCL and SL because we had just 2 non-blood samples to analyze from these subclasses. For models specifications, please refer to Table S1.

### 2.2. Classification Trees

The database was randomly splitted in two, as detailed in Section 4.4: 75% of the database (training set) was used to generate the classification trees, while the 25% (validation set) was used to validate the predictive models.

**Figure 1:**
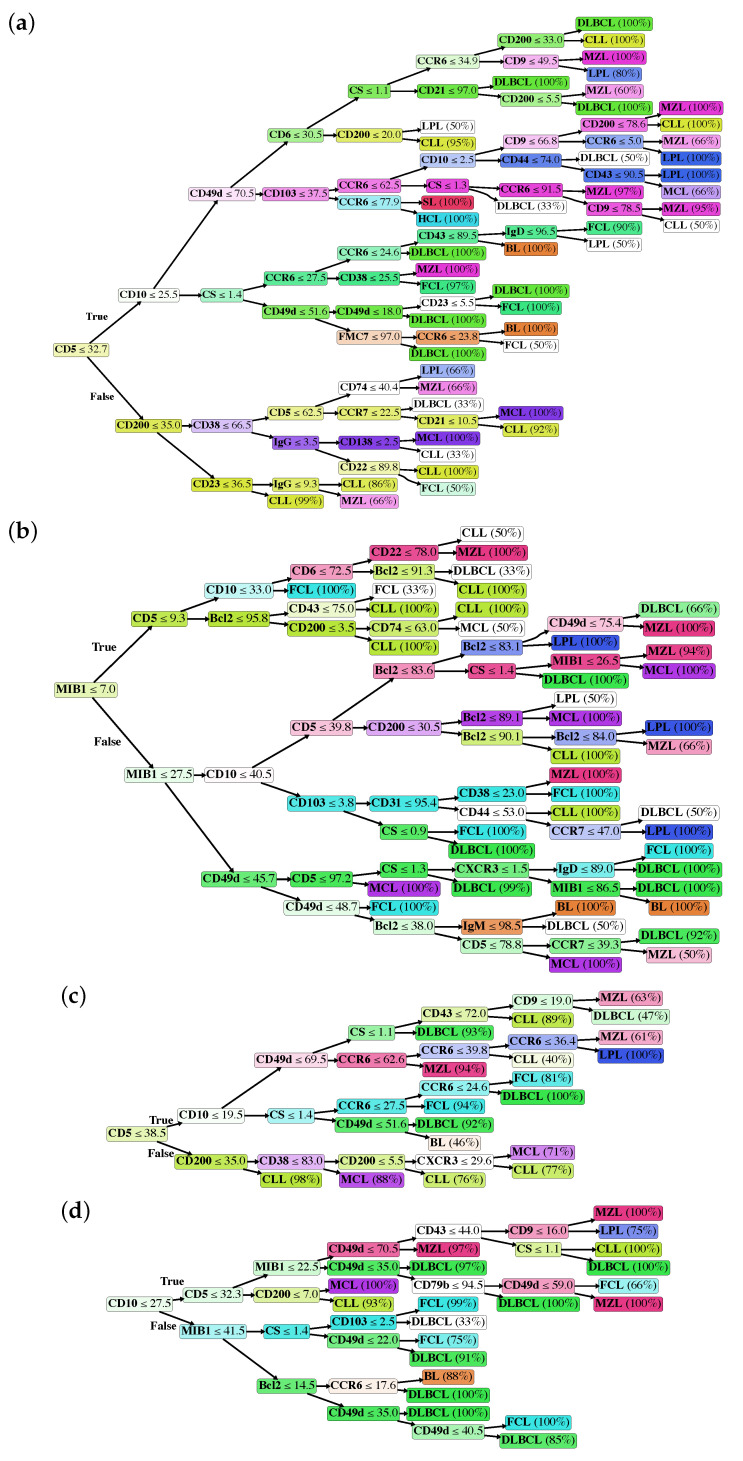
Classification trees of Model I (**a**), Model II (**b**), Model III (**c**), and Model IV (**d**). Values reported inside decision nodes represent threshold values. Percentage values reported in leaf nodes represent the prediction probability, i.e., how likely the achieved class is the correct one.

In particular, we generated the following classification trees, according to each model described in the previous paragraph: Model I (Figure 1a), Model II (Figure 1b), Model III (Figure 1c) and Model IV (Figure 1d). Classification trees are composed of a root node, i.e., the marker with the highest discriminative power, internal decision nodes with two outgoing edges, and leaf nodes, which represent the B-NHL class and the probability that the achieved class is the correct one.

### 2.3. Performances of the Predictive Models

Each generated tree has been tested on a validation set composed of samples that were not included in the training set. All results described below, therefore, refer to the evaluation of our predictive models against the validation set.

**Figure 2:**
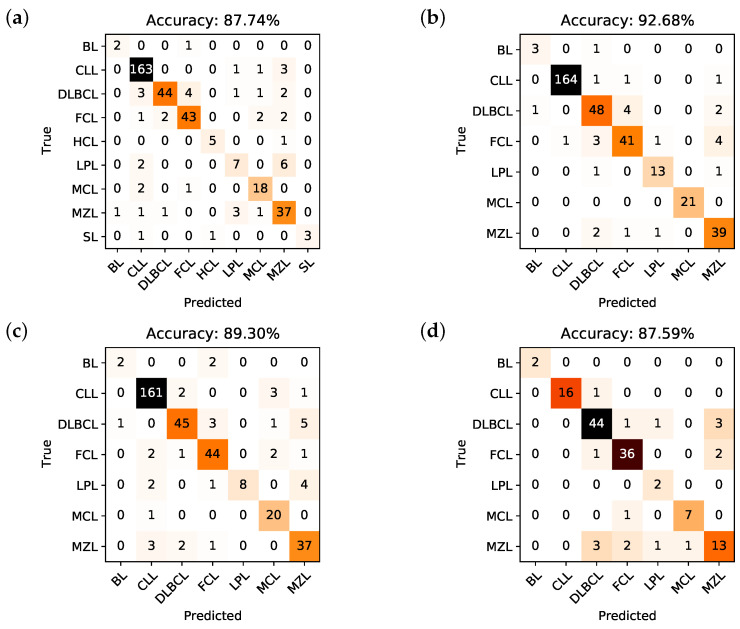
Confusion matrices and corresponding overall accuracies for the classifiers associated to Model I (**a**), Model II (**b**), Model III (**c**), and Model IV (**d**).

Here, we resort to confusion matrices in order to visualize the performance of each model. A confusion matrix is a way to visualize the predicted vs. the actual classification; in particular, the y-axis contains the true class the sample belongs to, while the x-axis contains the predicted class. In a classifier with a 100% accuracy, hence, all samples would be in the diagonal of the confusion matrix. The confusion matrices generated from the classification trees of Model I, Model II, Model III and Model IV are shown, respectively, in Figure 2a–d.

The accuracy, i.e., the number of correctly predicted samples divided by the total number of samples, that can be deduced from the confusion matrix of Model I is 87.74%, for Model II is 92.68%, for Model III is 89.3%, and for Model IV is 87.59%. It is important to underline that the obtained training accuracy for each model is respectively 85.97%, 91.27%, 86.67%, and 87.35%. This means that there is no overfitting, i.e., the predictive models are not too rigid nor calibrated on the training set, but they are rather capable of generalizing classification rules on new data quite effectively.

Going into details, Table 1 describes, respectively for Model I to IV, the accuracy, sensitivity, specificity, positive predictive value (PPV), and negative predictive value (NPV) for each subclass in every model. As it is possible to appreciate, the per class-accuracy is very high, and always higher than the global accuracy: this reflects the extremely good ability of the algorithms to discriminate a single class with respect to all the others (e.g., DLBCL vs not DLBCL), while it is obviously more difficult for the predictive models to understand which is the correct class (e.g., it is not DLBCL, it is FCL).

### 2.4. Analysis of the Predictive Models

**Figure 3:**
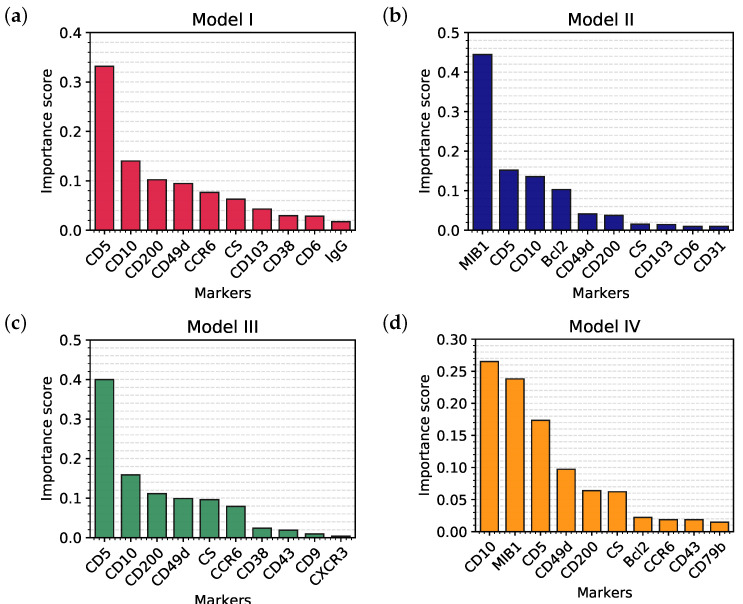
Top 10 markers in order of importance selected by Model I (**a**), Model II (**b**), Model III (**c**), and Model IV (**d**).

Unlike most ML or deep learning techniques, a classification tree allows the investigators to see the “rules” that the algorithm learned from the training set and then uses to classify the samples in the validation set. First of all, we can derive from the trees a classification of the markers according to their importance [23] in every model. Results are shown, respectively, in Figure 3a–d: the higher the importance, the higher the capability of discrimination, hence the higher the position inside the tree structure.

**Figure 4:**
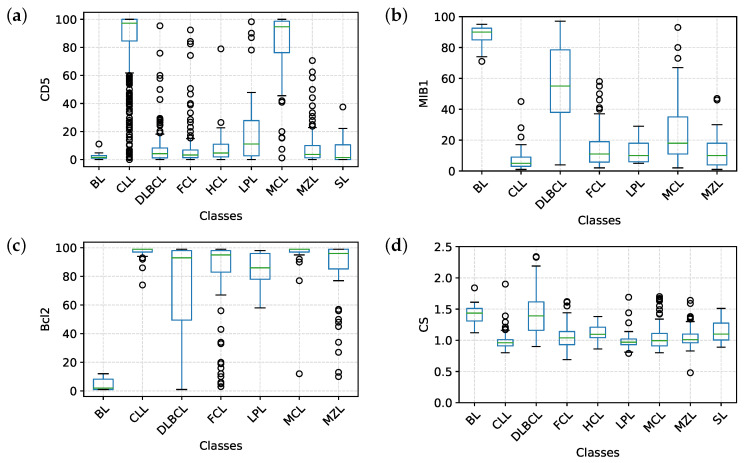
Distribution of CD5 (**a**), MIB1(**b**), Bcl2 (**c**) and CS (**d**) in all the samples of the database, versus B-NHL subclass. HCL and SL samples are not represented in MIB1 and Bcl2 plots because samples from these two classes have not been consistently evaluated for these two markers. Each box has a central mark that indicates the median of the distribution, whereas bottom and top edges of the box indicate the 25th and 75th percentiles, respectively. The whiskers extend to the most extreme non-outliers data points. Circle markers indicate outliers. CS stands for cell size.

The most important markers in each model represent the root and the first branches of each tree; this reflects their ability to clearly discriminate between different classes, as it can be appreciated in Figure 4a–c which represent the distribution of CD5, MIB1 and Bcl2 in the samples, divided by class. Finally, in order to compare our current clinical practice to the predictive models, we also evaluated the distribution (and hence the discriminatory power) of the cell size (CS), a simple parameter that is still very used in the FC evaluation of samples (Figure 4d).

## 3. Discussion

In this study, we applied ML techniques to our immunophenotype database in order to create four predictive models which could distinguish between several B-NHL, starting from samples with different sources.

First, we had to decide how to express immunophenotypes. We decided to use the percentage of positive cells, regardless of the fluorescence intensity, because we thought it would have been more standardized and less sensitive to the change of the reagents and/or flow cytometer employed. Moreover, it is extremely easy, and fits with our intent to create predictive models which could support decisions for future samples, possibly in different laboratories.

Afterwards, we had to decide which ML technique was more suitable for our purpose. We opted to use decision trees as classifiers (refer to Section 4.4) because we wanted to create an easy-to-interpret decision-support technique, i.e., a tool that could be manually inspected by the operators, guiding them in the evaluation of new samples; finally, we wanted to compare the behavior of the classifier to the human one, eventually confirming or suggesting improvements to our routine clinical practice.

A theoretical disadvantage of classification trees is that they define sharp thresholds for each marker: it is difficult to attribute any real meaning to these values, and they could potentially vary from one laboratory to another one. However, the fact that classification trees use thresholds and that a single marker is used along the tree with different thresholds in order to discriminate different B-NHL has indeed a biological meaning: it suggests that a marker is not just “positive” or “negative”, but a different percentage of cells expressing that marker could correlate with different B-NHL. In other words, it could be incorrect to use fixed thresholds to define a marker as positive or negative because (i) there could be different thresholds for the very same marker, indicating different B-NHL, and (ii) thresholds should vary from one marker to another one.

Finally, we had to deal with missing values: as our database spans from 2003 to 2019, different markers have been used according to the best knowledge and techniques available at that time. Moreover, the markers’ usage reflects the daily clinical practice, so that some markers are poorly utilized when a particular disease is suspected. This aspect is particularly important for MIB1 and Bcl2, as these two markers are mostly utilized in non-blood samples, and for HCL and SL classes, where we had especially blood samples. This issue led us to generate multiple models, as described in Section 2, Section 4.3, and in Table S1.

These analyses allowed us not only to evaluate our data in the most honest way, but also to compare the performances with and without MIB1 and Bcl2, two markers that have been increasingly used in our Laboratory with increasing satisfaction.

In Model I, the whole dataset was analyzed, but MIB1 and Bcl2 were removed. The tree is characterized by a good overall accuracy (87.74%) and a very good per class accuracy, ranging from 94.28% to 99.46%. However, accuracy is probably not the best indicator of the tree’s performance, as it is clear in the BL subclass, where sensitivity is 66.67% and accuracy 99.46%. The best indicators are probably PPV and NPV that range, respectively, from 58.33% to 100% and from 96.56% to 99.73%. Merging information from the confusion matrix and Table 1, we can say that Model I, on the whole, has high specificity and NPV, while sensitivity and PPV are suboptimal for some B-NHL subclasses. In particular, the model does not easily recognize BL, probably due to the low number of cases and to the fact that this model does not use MIB1. Moreover, the algorithm seems to confuse LPL with MZL, which is quite expected, considering that these 2 subclasses are both indolent lymphomas with largely overlapping features. The best recognized class in terms of sensitivity is CLL, likely due to the very high number of available samples the model could learn from. Analyzing the tree (Figure 1a), it is possible to appreciate that it recapitulates the classical cytofluorimetric approach to B-NHL classification: CD5 is the root that divides the top part (CD5 < 32.7%) from the bottom part (CD5 > 32.7%), where there are mainly MCL and CLL, distinguished by CD200. The top part of the tree is divided by CD10 in a middle part (CD10 > 25.5%), mainly occupied by DLBCL, FCL and BL, and in a top part (the classical CD5-ve, CD10-ve), which identifies primarily MZL, SL, HCL and LPL. However, there are some considerations worth to be noted:

1. As mentioned above, markers occur into the tree structure multiple times with different thresholds, applying more fine-grained selections as they approach leaf nodes.
2. Figure 3a clearly shows that, apart from CD5, CD10 and CD200, the most important parameters to classify B-NHL are CS and, intriguingly, CCR6, CD49d and CD6. CS is generally underused as a classifier in flow cytometry despite its common use as a gate [24,25]. To overcome the problem of the intrinsic variability of the forward scatter as a measure of the cell volume, due to different setups and different instruments, we calculated for each sample the ratio between the forward scatter of neoplastic B-cells and residual T-lymphocytes.
  CCR6 is the receptor of chemokine CCL20 and of beta-defensins. It is known to be expressed in B-lymphocytes of the mantle and marginal zone, and in their lymphomatous counterparts, but it is not routinely used to classify B-NHL, even though other groups have already reported that it is differentially expressed in various NHL [26]. CD49d is one of the strongest FC-based predictors of overall survival in CLL [27], but limited data are available on its expression in other NHL [28,29]. The importance of these two non-canonical markers, however, could have been overestimated due to missing values (Section 4.1) and to the filling methodology utilized; further studies are needed to confirm these results.
  Finally, the predictive model also selected CD6, a CD5-related antigen: they are both scavenger-receptor glycoproteins, expressed on normal T-cells and a small subset of normal B-cells [30] and generally highly expressed in CLL and MCL. According to this model, CD6 seems to identify those rare forms of CLL which express low levels of CD5, probably functionally substituting it.
3. Some markers which are generally used in a particular setting can have a role in classifying other B-NHL: CD200 and CD23, for example, which are basically used to distinguish CLL from MCL, seem to be differentially expressed in other B-NHL, such as DLBCL or MZL.

In Model II, MIB1 and Bcl2 were included, but we had to remove HCL and SL from the analyses, as samples from these two subclasses had not been consistently screened for these two markers. The introduction of MIB1 and Bcl2 in the analysis significantly enhances the overall accuracy (92.68%), with major improvements in sensitivity, specificity, PPV and NPV of every subclass, which are all extremely high.

The strong discriminatory power of MIB1 and Bcl2 is evident in Figure 4b,c, justifying the great impact of these two markers on performances. Accordingly, MIB1 is the most important marker in this model (Figure 3b) and it represents the root of the tree. MIB1 divides the tree in three parts (Figure 1b): the top, with a very low MIB1 level (<7%), includes the majority of CLL, some FCL, some MZL, and sporadic cases of MCL. The bottom part of the tree (MIB1 levels > 27.5%) includes all BL, the majority of DLBCL, some MCL and sporadic cases of FCL and MZL. The intermediate part contains the majority of FCL, MCL, LPL, and MZL, sporadic cases of DLBCL and CLL. Interestingly, MIB1 recurs along the tree, with different thresholds, to furtherly discriminate specific classes.

MIB1 identifies Ki-67, i.e., a nuclear antigen which is expressed by proliferating cells. Ki-67 is known to be differentially expressed in B-NHL, being highly expressed in aggressive B-NHL [31,32], and to have a strong prognostic impact [33]. However, data on MIB1/Ki-67 mainly derive from IHC, while its evaluation in FC has still limited evidence.

Bcl2 is an antiapoptotic protein; while its rearrangement is typically seen in FCL, some DLBCL and double/triple hit lymphomas, Bcl2 is also widely and differentially expressed in other B-NHL lacking t(11;14), including CLL and MZL [34,35,36,37]. The expression of Bcl2, and not only the translocation, has a prognostic impact on many B-NHL, especially in the so-called double expressor DLBCL [38,39]. Again, the vast majority of these data derive from IHC, while FC data would be highly desirable.

Model II surely outperforms Model I, but the starting dataset is not the same: in order to evaluate the net benefit of MIB1 and Bcl2, we have to compare Model II with Model III. Although the benefit in terms of overall accuracy seems to be modest (92.68% in Model II vs 89.3% in Model III), we can appreciate in the subclass analysis how sensitivity and PPV drop when MIB1 and Bcl2 are removed (Table 1). In summary, Model II outperforms Model III, whose tree and characteristics are very similar to Model I.

Finally, in order to demonstrate that the importance of MIB1 and Bcl2 was not an artifact due to the filling methodology utilized, we analyzed non-blood samples only, which are for the most part characterized with these two markers (Model IV). This resulted in a significant reduction of available samples, from 1465 to 548, so that we could not expect the performances of this algorithm to be comparable to other models. However, the overall accuracy is still 87.59%; more importantly, MIB1 still plays an extremely important role in the classification tree, being second only to CD10 (Figure 3d), and recurring multiple times in the tree (Figure 1d); also Bcl2 is still between the most powerful and discriminatory markers, just like in Model II (Figure 3b). Model II and Model IV have many similarities, as can be noted from the most important marker plots for each tree (Figure 3b vs. Figure 3d); differences can be explained, for the most part, by the different starting datasets (Model II, for example, is much more enriched with CLL compared to Model IV). This clearly demonstrates that the importance of MIB1 and Bcl2 is real, excluding any artifactual effect.

The last point to highlight is that, while the database included samples with an extended characterization, the classification trees only evaluate a very limited number of markers. Indeed, if a hypothetical FC operator decided to follow the tree structure node-by-node, he could arrive at a leaf node, i.e., at the diagnosis, with only 8 markers in the worst case scenario.

Our study suffers from some limitations, mainly due to its retrospective nature. Cases were classified according to the 2008 WHO classification. The main consequence is that B-NHL double /triple hit lymphomas were classified as DLBCL; we could not correct this classification, because we did not have molecular or cytogenetic data in the vast majority of DLBCL samples, but we are planning to introduce this new class in our future work.

Second, not every sample is characterized for every marker, prompting us to generate multiple models; with this strategy we tried to reduce any bias due to the preprocessing on missing values, but our results need to be validated in a prospective study with no, or limited, missing values.

Third, this is a monocentric study; our database, though, spans from 2003 to 2019, different markers as well as different machines have been utilized, and different operators have been analyzing data, partially mitigating the single center effect.

Finally, our method is not totally automated: data have to be analyzed manually, with the operator still in charge of data preprocessing, gating and results extraction (percentage of cells expressing each marker). In the setting of totally automated techniques, previous works [20,21,22] seem to have achieved very good preliminary results, even though they only consider blood samples, thus excluding B-NHL that typically involve lymph nodes and tissues, such as DLBCL or BL, while including many non-neoplastic samples. These automated analyses try to substitute the human role in a routine setting (PB and BM analyses of suspected B-NHL samples), utilizing canonical markers chosen from the human experience; investigators cannot understand how the predictive model yields results, and they have to accept them for good. Rather than a limitation of our work, this is a choice we made at the beginning of our research: we decided to create an easy-to-interpret tool, that the operator could visualize and literally follow, which could support diagnostic decisions in the routine clinical activity, possibly optimizing the choice of the markers to utilize.

## 4. Materials and Methods

### 4.1. Flow Cytometry

FC analyses were performed on blood and non-blood samples. Blood samples included PB and BM aspirates; non-blood samples included lymph nodes and tissue biopsies or FNA, pleural and peritoneal effusions, liquor and bronchoalveolar lavage.

All PB and BM samples were homogeneously treated. After counting the cells in the hemocytometer, cell concentration was adjusted (if greater than) at $20\times 10^{6}$/mL with RPMI 1640 containing 10% FBS. Fifty to 100 μL of samples were directly added to a monoclonal antibody mixture and incubated 15–30 min at room temperature; red blood cells were lysed with ammonium chloride solution (10 min at room temperature), washed with PBS/BSA 0.5%, resuspended and immediately acquired. A cell suspension of tissue samples was obtained by mechanical disaggregation, immediately counted and evaluated for cell viability in the hemocytometer. If viability was less than 70% and/or the total number of viable cells was greater than $10\times 10^{6}$, mononucleated cells were then purified by density gradient; otherwise, death cells were excluded from analysis using 7AAD staining. Cells were then washed twice with RPMI 1640 + FBS 10%, and resuspended in the same medium at 5–$20\times 10^{6}$/mL. Fifty to 100 μL of samples were directly added to a monoclonal antibody mixture, incubated 15–30 min at room temperature, washed twice with PBS/BSA 0.5%, resuspended and immediately acquired. The staining for intracellular antigens was performed after surface staining using Fix & Perm™ reagents and protocol (Invitrogen—Carlsbad, CA, USA). To obtain an optimal intracellular staining of cells from blood samples, when required, mononuclear cells were purified on density gradients from PB or BM.

This study is a 16-year retrospective study: over this period of time, the instruments and reagents used have been changed several times, adapting to technological evolution. The oldest flow cytometric analyses (2003–2006) were performed with a 2-laser and 4-color FACSCalibur BD (Becton Dickinson—Milan, Italy); in 2006–2007, we used an FC500 Beckman Coulter (Beckman Coulter—Milan, Italy) with 2 lasers and 5 colors, followed (2007–2010) by a CyAn (Dako—Santa Clara, CA, USA) with 3 lasers and 8 colors; finally, analyses from 2010 to 2019 were performed with a 3-laser and 10-color Navios Beckman Coulter. Similarly, most of the antibodies used were initially only available with FITC, PE, PerCP and APC fluorochromes; over time, monoclonal antibodies conjugated with Pacific Blu, APC-Alexa700, APC-Alexa750, PE-Cy5 / PE-Cy5.5 and PE-Cy7 have been added.

**Figure 5:**
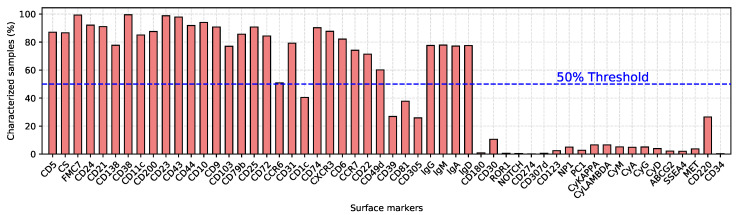
List of surface markers associated with the percentage of samples characterized for each marker. The blue dotted line indicates the threshold (50%) above which markers were considered.

Unlike all the technical changes described above, the analytic strategy of the samples remained the same, although initially it required more tubes and more time, being limited by the available instrument (please refer to Supplementary Figure S1). Basically, a first series of tubes, recently reduced to a 12 antibodies-8 colors single tube, is carried out in order to identify the monoclonal cell population, i.e., the backbone markers which guarantee a purity of the monoclonal population greater than 95% in 97% of cases, and greater than 90% in the remaining samples. Figure 5 contains the list of surface markers utilized to analyze the samples, together with the percentage of samples that were characterized for that marker.

The intracellular markers analyses (Bcl2 and MIB1) were performed in most cell suspensions from tissue samples, but only sporadically in PB and BM samples. This is due to a standardization of the method used for intracellular analysis, which requires mononuclear cells purification with density gradient, a procedure that is not routinely used for PB and BM samples. Furthermore, the intracellular antigens analysis for the majority of PB and BM samples was not clinically required. Table S2 provides the list and the characteristics of surface and intracellular markers employed. The total number of events acquired for each tube was influenced by the flow cytometer available at the time of the analysis, ranging from a minimum of 10,000 total events per tube to 500,000 total events.

The analysis of each single tube was performed with the software supplied with the corresponding instrument (CellQuest Pro for FACSCalibur, CXP for FC500, Summit for CyAn and Navios Software for Navios). All the reported values refer to the percentage of positive neoplastic cells for each marker, except for the CS, calculated as the mean fluorescence intensity (MFI) ratio between forward scatter of neoplastic B-cells and residual T-lymphocytes. Isotypic controls for each fluorochrome were used in all the samples analyzed and, if not available, the internal control of non-neoplastic cells (negative for the marker under examination) was used to define the position of the markers.

### 4.2. Data Collection

The data used in this study were collected by the Laboratory of Immunopathology at the A.O. Ordine Mauriziano, Turin (Italy). The dataset comprises 1547 de-identified immunophenotypic profiles of clonal B-cells lymphoproliferative disorders, with the exclusion of Plasma Cell disorders, deriving from tissue biopsies, liquor, effusions, PB or BM samples, collected from 2003 to 2019. No personal information was included in the dataset. Each diagnosis of lymphoma or leukemia was supported by case history, cytology, histopathology, immunohistochemistry, flow cytometry, cytogenetic analyses, and molecular biology available at the time of diagnosis. Each sample was evaluated by a first expert hemato-pathologist and then reviewed by a second one. The final diagnosis was decreed when pathologists had received cytogenetic and molecular biology analyses (if necessary), and after a consultation with the clinical hematologist. All the cases were classified according to the 2008 WHO classification of lymphoid neoplasms, despite the 2016 update, because of the impossibility to revise and update old cases; in particular, we could not distinguish between DLBCL and double or triple hit lymphomas due to the lack of specific cytogenetic analyses in old samples. Data collection and analyses were approved by the Ethical Committee of the A.O. Ordine Mauriziano (protocol number 0044740, issued on 30 April 2019)

### 4.3. Data Preprocessing

We started from a database containing 1547 samples, classified according to the 2008 WHO classification of lymphoid neoplasms [1]. Initially, we had 20 classes; however, B-cell prolymphocytic leukemia and primary effusion lymphoma were excluded from the analyses because of the paucity of available samples (2 per class). Moreover, 61 non-CLL type monoclonal B-cell lymphocytosis (MBL) samples were excluded, as they could not be easily categorized; 1 sample was removed because very poorly characterized; finally, 16 samples could not be surely classified and were removed. In order to increase the number of samples for each B-NHL subclass, we chunked together affine diagnosis according to the association rules described in the following.

- **HCL**: Hairy cell leukemia
- **SL**: Splenic lymphoma/leukemia unclassifiable
- **LPL**: Lymphoplasmacytic lymphoma
- **FCL**: Follicular cell lymphoma
- **MCL**: Mantle cell lymphoma
- **BL**: Burkitt lymphoma
- **CLL**: Chronic lymphocytic leukemia, including small lymphocytic lymphoma and high-count CD5+ MBL (CLL-type MBL).
- **MZL**: Marginal zone lymphoma, including splenic marginal, nodal marginal, and MALT lymphoma.
- **DLBCL**: Diffuse large B-cell lymphoma, including T-cell/histiocyte-rich large B-cell lymphoma, primary DLBCL of the CNS, primary cutaneous DLBCL, and double/triple hit lymphomas.

CLL-type high-count MBL, i.e. with a PB CLL count of >0.5 and <5 $\times 10^{6}$/mL, were included in the CLL class. MBL, in fact, was not classified as a clear entity in the 2008 WHO classification, while the 2016 WHO classification states that high-count MBL have very similar phenotypic and genetic/molecular features to Rai stage 0 CLL, with just a higher percentage of IgHV-mutated cases [1,2].

At the end of this data preprocessing, we obtained a dataset of 1465 samples, categorized into 9 different classes (Table 2).

Each sample was extensively characterized. However, utilized markers have changed over the 16-year period during which data were collected. As a result, recently analyzed samples include markers that became of interest in the last few years, whereas early samples include some markers that are currently no longer used. This has led to a database with several missing values. To overcome this problem, we adopted three different strategies. First, surface markers with a 50% or higher missing-value rate were completely discarded from the database (see Figure 5). Second, class-wise mean values were used to impute unavailable data [40]. No additional selection has been carried out on the available markers. Indeed, we wanted to rely on the ML algorithm for selecting the most significant and representative set of markers for the entire database. Third, we generated multiple models taking into account the missing values of intracellular markers. Table S1 shows more detailed model specifications.

### 4.4. Predictive System

Each predictive model is composed of a single classification tree, a supervised learning technique introduced in [41]. A classification tree is a rooted and directed acyclic graph (DAG) defined as Γ=(Φ∪D∪Θ∪E). The root node ϕ∈Φ with an in-degree 0 represents the most effective classification rule. Terminal nodes θ∈Θ with an out-degree 0 represent the class to which a generic input sample is associated. Each decision node d∈D implements a threshold (T${}_{h}$) comparison with an FC marker. The result of such comparison fires one of the two outgoing edges e${}_{f}$, e${}_{t}$∈E, enabling the connection towards nodes at the lower levels: if a marker m${}_{k}$≤ T${}_{h}$ then the true branch e${}_{t}$ is active, otherwise the false branch e${}_{f}$ is active. Depending on the values assumed by FC markers of a given sample, only one root-to-leaf path is activated.

Classification trees have been obtained with a script developed in *Python* leveraging the open-source libraries *Pandas* [42], for data preprocessing, and *SciKit learn* [43], for training and validation. For each classification tree, the training algorithm has been applied on a subset of the dataset containing the 75% of the samples, whereas validation has been carried out on the remaining 25% of the population. Since training and validation samples were randomly drawn from the entire dataset, and since available labels are considerably unbalanced, we ensured that all classes were represented in both training and validation sets by means of a stratified random selection [44]. The parameters of each classification tree were determined according to a grid search analysis, i.e., a thorough exploration of specific classification tree configurations by means of empirical evaluations. More in particular, for each classification tree, the following parameters have been included in the grid search analysis:

- Maximum depth, with values ranging from 6 to 12;
- Minimum number of samples per split, with values ranging from 2 to 10;
- Minimum number of samples per leaf node, having values in the range 1, 10;
- Maximum number of features criterion (*sqrt*, *log2*, or unconstrained);
- Optimal split selection criterion (*best* or *random*);
- Split criterion (*Gini impurity* or *information gain*).

The grid search has been combined with the *k*-fold cross-validation [45], where *k* has been set according to the number of unique labels. It is important to notice that *k*-fold has been carried out accounting samples of the training set only. The best model was then selected according to the highest accuracy, and then evaluated against the validation set.

Two main reasons led us to choose classification trees as a preferred tool. First, they intrinsically perform a selection on the available features, a property that has its roots in the training algorithm [46]. Indeed, at each step of the learning process, the best split feature, i.e., the one that performs the most homogeneous separation of the training samples, is determined. The training algorithm considered in this paper relies on the Gini impurity [47] or on the information gain [48] to quantify the amount of entropy of each possible split. If we assume a dataset having N different labels, the Gini impurity is defined as the summation of the probability $p_{n}$ of a sample being labeled n, times the probability of a mistake in labelling that item, i.e., 1 – $p_{n}$, where n ∈ N. By evaluating the Gini impurity associated to each feature, the training algorithm selects the most significant feature for each step of the learning process. Ideally, a good split should be able to separate the training dataset into disjoint sets, where samples in each set belong to one class only. Since such a partitioning is performed through recursive splits of the training dataset, an a priori stopping criteria is usually defined. The objective is to avoid the tree to grow indefinitely, thus leading to extremely deep classification trees having splits that are completely homogeneous but that may have insignificant sample sizes, a phenomenon known as overfitting. This suggests that early stopping criteria guarantee a compact and shallow tree structure, implying that only a subset of the available features, i.e., the most significant ones, are selected.

The second motivation for using classification trees concerns the interpretability of the obtained predictive models. It is well known indeed that more powerful learning techniques do not clearly expose the rules learnt during training, thus leading to predictive models that are mostly used as black-box systems. On the contrary, our objective was to clearly identify what those learnt rules are, and also how those rules are connected with each other. In this regard, we are convinced that classification trees represent a perfect trade-off between quality-of-results and intelligibility of the model structure.

## 5. Conclusions

In this work, we generated multiple, clinically applicable predictive systems, with good to optimal performances, that can be applied to every biological material involved in lymphoproliferative diseases, from blood to lymph nodes or pleural effusions.

This confirms that FC has great potential in B-NHL diagnosis and classification as (i) it can give reliable information when histology is not available (FNA samples, B-NHL involving difficult-to-biopsy sites, and so on) and (ii) it can give complimentary information to histology, and in a very timely manner.

By evaluating the decision trees node-wise, the FC operator can define the most likely B-NHL subclass with just a few steps, therefore limiting the amount of markers needed to achieve a diagnosis. This could potentially lead to an optimization of the workflow in FC laboratories. Interestingly, some of the markers selected by the predictive models are not routinely used to classify B-NHL, such as CD49d and CCR6. Bcl2 and MIB1 have shown strong discriminatory power; further prospective studies are needed to confirm if their introduction in the routine evaluation of samples could improve FC diagnostic accuracy in B-NHL. Finally, we demonstrated that the different levels of marker expression often correlate with different NHL, suggesting that defining a marker as positive or negative based on a fixed threshold could represent a loss of information.

Further prospective multi-center studies will be fundamental to verify our insights and to refine our models.

## Figures and Tables

**Table 1:** Accuracy, sensitivity, specificity, positive predictive value (PPV), and negative predictive value (NPV) for each B-NHL subclass for Model I to IV. All values are percentages.

B-NHL	Accuracy	Sensitivity	Specificity	PPV	NPV
**Model I**					
**BL**	99.45	66.67	99.73	66.67	99.73
**CLL**	95.90	97.01	94.96	94.21	97.42
**DLBCL**	96.18	80.00	99.04	93.62	96.56
**FCL**	96.45	86.00	98.10	87.76	97.79
**HCL**	99.45	83.32	99.71	83.32	99.71
**LPL**	96.45	46.67	98.57	58.32	97.75
**MCL**	97.81	85.70	98.54	78.26	99.12
**MZL**	94.28	84.09	95.67	72.54	97.78
**SL**	99.45	60.00	100.00	100.00	99.45
**Model II**					
**BL**	99.43	75.00	99.71	75.00	99.71
**CLL**	98.87	98.20	99.46	99.39	98.42
**DLBCL**	95.76	87.26	97.32	85.70	97.65
**FCL**	95.76	82.00	98.03	87.23	97.07
**LPL**	98.87	86.67	99.40	86.67	99.40
**MCL**	100.00	100.00	100.00	100.00	100.00
**MZL**	96.62	90.70	97.43	82.98	98.70
**Model III**					
**BL**	99.15	50.00	99.71	66.67	99.43
**CLL**	96.06	96.40	95.73	95.26	96.76
**DLBCL**	95.76	81.81	98.32	90.00	96.71
**FCL**	96.34	88.00	97.70	86.26	98.03
**LPL**	98.03	53.32	100.00	100.00	97.98
**MCL**	98.03	95.23	98.20	76.92	99.70
**MZL**	95.20	86.04	96.46	77.07	98.04
**Model IV**					
**BL**	100.00	100.00	100.00	100.00	100.00
**CLL**	99.26	94.12	100.00	100.00	99.17
**DLBCL**	92.70	89.79	94.31	89.79	94.31
**FCL**	94.89	92.31	95.92	90.00	96.90
**LPL**	98.54	100.00	98.51	50.00	100.00
**MCL**	98.54	87.50	99.21	87.50	99.21
**MZL**	91.23	65.00	95.73	72.21	94.12

BL—Burkitt Lymphoma, B-NH—B-cell Non-Hodgkin Lymphomas, CLL—Chronic Lymphocytic Leukemia, DLBCL—Diffuse Large B-cell Lymphoma, FCL—Follicular Cell Lymphoma, HCL—Hairy Cell Leukemia, LPL—Lymphoplasmacytic Lymphoma, MCL—Mantle Cell Lymphoma, MZL—Marginal Zone Lymphoma, NPV—Negative Predictive Value, PPV—Positive Predictive Value, SL—Splenic Lymphoma.

**Table 2:** Distribution of samples in B-NHL categories. Samples are furtherly divided according to their origin: blood samples (PB/BM) and non-blood samples (lymph nodes and tissue biopsies or FNA, pleural and peritoneal effusions, liquor and bronchoalveolar lavage).

B-NHL Category	Total Samples	Blood Samples	Non-Blood Samples
CLL	670	602	68
FCL	199	43	156
SL	19	17	2
MZL	174	94	80
DLBCL	220	25	195
LPL	60	53	7
MCL	83	51	32
HCL	26	26	0
BL	14	4	10

## Supplementary Materials

The following are available at https://www.mdpi.com/2072-6694/12/6/1684/s1, Figure S1: Flow cytometric gating strategy in different sample types/lymphoma entities, Table S1: Model specifications, Table S2: List and characteristics of surface and intracellular markers employed.
