# Supplementary material for: A Clinically Applicable Approach to the Classification of B-Cell Non-Hodgkin Lymphomas with Flow Cytometry and Machine Learning
Source: Cancers (Basel). 2020 Jun 24;12(6):1684. doi: 10.3390/cancers12061684 (PMC7352227; doi:10.3390/cancers12061684)
Supplement: Supplementary file 1 [file cancers-12-01684-s001.pdf]

Supplementary Material: A Clinically Applicable Approach to the Classification of B-cell Non-Hodgkin Lymphomas with Flow Cytometry and Machine Learning

Valentina Gaidano, Valerio Tenace, Nathalie Santoro, Silvia Varvello, Alessandro Cignetti , Giuseppina Prato, Giuseppe Saglio , Giovanni De Rosa, and Massimo Geuna

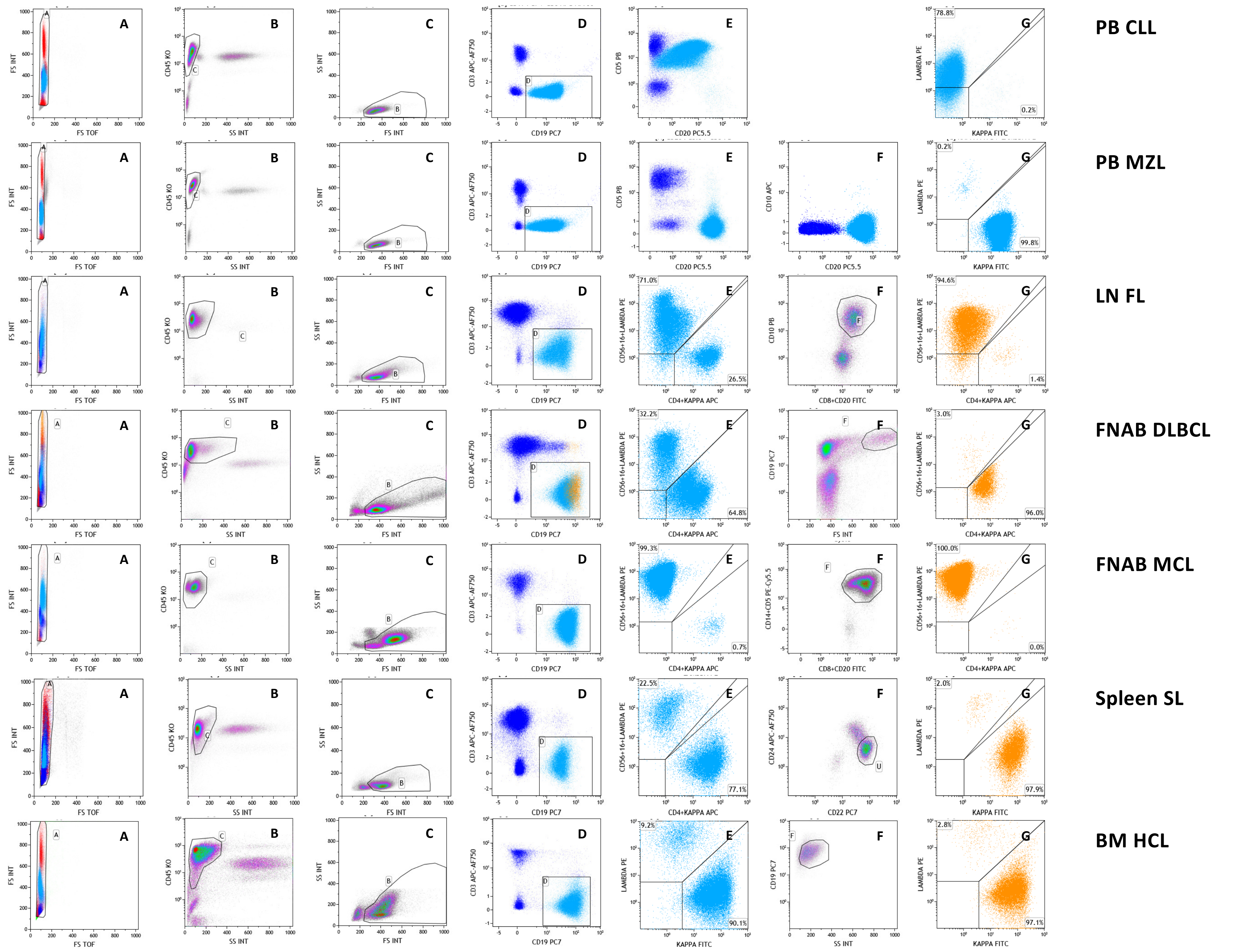

Figure S1. Flow cytometric gating strategy in different sample types/lymphoma entities. The sequence of gate is aimed to obtain the greatest purity of neoplastic cells as identified by means of the clonal restriction of immunoglobulin light chains (kappa or lambda). In all cases the first four plots (A, B, C and D) follow an identical scheme: plots A are FS TOF (Forward Scatter Time Of Flight) vs FS INT (Forward Scatter Intensity) and the gate A is drawn to exclude doublets. Plots B, activated on gate A, are SS INT (Side Scatter Intensity) vs CD45 and the gate C is drawn around CD45 positive cells with low SS, to exclude granulocytes (CD45+/SS^{high}) and unlysed/ghost erythrocytes (CD45-/SS^{low}). Plots C, activated on the Boolean gate [A AND C], are FS INT vs SS INT where all the cells identified with the previous gates are further gated (gate B) to exclude debris (low FS and low SS). Plots D, activated on the Boolean gate [A AND C AND B], are CD19 (total B cells) vs CD3 (total T cells) and the gate D is drawn to select all CD19 positive cells. PB B-CLL (Peripheral Blood, B Chronic Lymphocytic Leukemia): plot E (gated on [A AND C AND B]) shows the typical co-expression of CD20 (low intensity) vs CD5 of B –CLL CD19 positive B lymphocytes, colored in light blue; plot G (activated on Boolean gate [A AND C AND B AND D]) shows the expression of kappa and lambda light chains on CD19 positive cells: almost all B cells are clonally restricted for lambda light chain (with low fluorescence intensity). PB-MZL (Peripheral Blood, Marginal Zone Lymphoma, leukemic phase): plots E and F (gated on [A AND C AND B]) show the negativity of leukemic cells for both CD5 and CD19 respectively. Plot G (activated on Boolean gate [A AND C AND B AND D]) shows the expression of kappa and lambda light chains on CD19 positive cells: 99.8% of B cells are clonally restricted for kappa light chain (with intermediate fluorescence intensity). LN FL (Lymph Node, Follicular Lymphoma): Plot E (gated on [A AND C AND B AND D]) shows the kappa/lambda distribution (with a ratio of 1/2.73) on the total CD19+ B cells. Plot F (gated on [A AND C AND B AND D]) shows the presence of a population of CD20+high, CD10+ cells that are gated in region F. The plot G (gated on [A AND C AND B AND D AND F]) shows the expression of kappa and lambda light chains on CD19+, CD20+high, CD10+ cells: 94.6% of B cells (virtually 98.5%) are clonally restricted for lambda light chain. FNAB DLBCL (Fine Needle Aspiration Biopsy, Diffuse Large B Cell Lymphoma): Plot E (gated on [A AND C AND B AND D]) shows the kappa/lambda distribution (with a ratio of 2/1) on the total CD19+ B cells. Plot F (gated on [A AND C AND B AND D]) shows the presence of a minor population of CD19+ B cells with a high FS (Large B cells, gated on F). The plot G (gated on [A AND C AND B AND D AND F]) shows the expression of kappa and lambda light chains on the CD19+ large B cells that are clonally restricted for kappa light chain (96% of expression at low intensity). FNAB MCL (Fine Needle Aspiration Biopsy, Mantle Cell Lymphoma): Plot E (gated on [A AND C AND B AND D]) shows the kappa/lambda distribution on the total CD19+ B cells with less than 1% of kappa positive cells. Plot F (gated on [A AND C AND B AND D]) shows that almost all CD19+ cells co-express CD5 and CD20, both at high fluorescence intensity, gated on F. The plot G (gated on [A AND C AND B AND D AND F]) shows the expression of kappa and lambda light chains on the CD19+, CD20+, CD5+ B cells that are clonally restricted for lambda light chain at 100% with high fluorescence intensity. Spleen SL (Spleen, Splenic Lymphoma): Plot E (gated on [A AND C AND B AND D]) shows the kappa/lambda distribution on the total CD19+ B cells with a kappa:lambda ratio of 3.4:1. Plot F (gated on [A AND C AND B AND D]) shows the expression of CD22 and CD24 on the total B cells, a discrete population of CD22+high/CD24+low cells is clearly identified and gated on F. The plot G (gated on [A AND C AND B AND D AND F]) shows the expression of kappa and lambda light chains on the CD19+, CD22+high, CD24+low B cells that are clonally restricted for kappa light chain with a purity of 97.9%. BM HCL (Bone Marrow, Hairy Cell Leukemia): Plot E (gated on [A AND C AND B AND D]) shows the kappa/lambda distribution on the total CD19+ B cells with 90.1% of kappa B cells. Plot F (gated on [A AND C AND B AND D]) shows the characteristic high SS of HCL B cells that are gated on F. The plot G (gated on [A AND C AND B AND D AND F]) shows the expression of kappa and lambda light chains on the CD19+, SS high B cells that are clonally restricted for kappa light chain with a purity of 97.1%.

Table S1. Model specifications. The column Samples reports the number of samples in the dataset, Samples/Class reports the number of samples for each class, Samples/TS and Samples/VS report the same information for the adopted training (TS) and validation (VS) datasets, whereas Markers describes the markers used. SM indicates surface markers above the 50% threshold, as in Figure 5.

Model	Samples	Classes	Samples/Class	Samples/TS	Samples/VS	Markers
**I**	1465	BL	14	11	3	SM
		CLL	670	502	168	
		DLBCL	220	165	55	
		FCL	199	149	50	
		HCL	26	20	6	
		LPL	60	45	15	
		MCL	83	62	21	
		MZL	174	130	44	
		SL	19	14	5	
**II**	1420	BL	14	10	4	SM, Bcl2, MIB1
		CLL	670	503	167	
		DLBCL	220	165	55	
		FCL	199	149	50	
		LPL	60	45	15	
		MCL	83	62	21	
		MZL	174	131	43	
**III**	1420	BL	14	10	4	SM
		CLL	670	503	167	
		DLBCL	220	165	55	
		FCL	199	149	50	
		LPL	60	45	15	
		MCL	83	62	21	
		MZL	174	131	43	
**IV**	548	BL	10	8	2	SM, Bcl2, MIB1
		CLL	68	51	17	
		DLBCL	195	146	49	
		FCL	156	117	39	
		LPL	7	5	2	
		MCL	32	24	8	
		MZL	80	60	20	

Table S2. List and characteristics of surface and intracellular markers employed.

Name (CD)	Alternative name	Cellular distribution	Function	Main use in B-cell lymphoma
**CD1c**	M241, R7, T6	cortical thymocytes, Langerhans cells, DC, B subset	Non peptide antigen presentation with β2-microglobulin to T-cell receptors on NKT cells.	Differential expression in chronic lymphoproliferative disorders (a). Upregulated in MALT lymphoma (b)
**CD5**	T1, Tp67, Leu-1, Ly-1	thymocytes, T, B subset, B-CLL	Regulates T-cell: B-cell interactions. Interacts with CD72	Marker of CLL/SLL and MCL
**CD6**	T12, TP120	thymocytes, T, B subset	Thymocyte development, a potential market of T-cell activation	Marker of CLL/SLL and MCL (CD5 surrogate)
**CD9**	p24, DRAP-1, MRP-1	pre-B, eosinophils, basophils, platelets, activated T cells	Cell adhesion and migration, platelet activation and aggregation	Inversely correlated with B lymphoma progression (c)
**CD10**	CALLA, NEP, gp100, EC 3.4.24.11, MME	B precursors, T precursors, neutrophils	Zinc-binding metalloproteinase, regulates B-cell growth	Marker of FL and GC-DLBCL
**CD11c**	Integrin aX, p150,95, AXb2, CR4	Dendritic cells, myeloid cells, NK, B, T subset	Cell adhesion, binds CD54, fibrinogen and iC3b	Marker of HCL and SMLZ (d)
**CD21**	CR2, EBV-R, C3dR	B cells, Follicular dendritic cells , T subset	Signal transduction (complex with CD19 and CD81, BCR coreceptor). Receptor for complement components C3Dd and iC3b as well as the Epstein-Barr virus (EBV) glycoprotein gp350/220,	Marginal zone B cell marker (e) and prognostic indicator in DLBCL (f)
**CD22**	BL-CAM, Siglec-2	B cells	Adhesion, B–T cell interactions	B cell marker
**CD23**	FceRII, B6, BLAST-2, Leu-20	B, activated macrophages, eosinophils, , Follicular dendritic cells , platelets	Low affinity receptor for IgE, ligand for CD19, CD21 and CD81	CLL/SLL marker, Matutes scoring system (g)
**CD24**	BBA-1, HSA	Thymocytes, erythrocytes,	GPI-linked receptor for signal	B cell differentiation marker

		granulocytes, B cells	transduction, regulation of B-cell proliferation and differentiation	
**CD25**	Tac antigen, IL-2Ra, p55, TCGFR	T activated cells, B activated cells, lymphocyte progenitors, Treg cells	IL-2Rα, associates with IL-2Rβ and γ to form high-affinity complex for IL-2, signal transduction	HCL marker
**CD31**	PECAM-1, endoCAM, Platelet endothelial cell adhesion molecule, PECA1	Monocytes, platelets, granulocytes, endothelial cells, lymphocyte subsets	Adhesion, signal transduction. CD38 receptor	heterogeneous expression pattern related to B cell lymphoma histogenetic derivation (h)
**CD38**	ADP-ribosyl cyclase, T10, Cyclic ADP-ribose hydrolase 1	Variable levels on majority of hematopoietic cells, high expression on plasma cells, B and T activated cells	Ecto-ADP-ribosyl cyclase, cell activation, adhesion, proliferation	Plasma cell marker, prognostic marker in CLL/SLL (i)
**CD39**	Ectonucleoside triphosphate diphosphohydrolase 1 (ENTPD1), ATPdehydrogenase, NTPdehydrogenase-1	B cells, NK cells, macrophages, Langerhans cells, Dendritic cells, Treg cells, T activated cell subset	Removal of extracellular ATP by ectozyme (ecto-apyrase), immune response support to anti-inflammatory conditions	Differentiation of non-GCB DLBCL from BL and FL (j)
**CD43**	Sialophorin, Leukosialin, Galactoglycoprotein, SPN	leukocytes, except resting B, platelets	Anti-adhesion. Binds CD45 to mediate adhesion	Coexpressed in the majority of CD5+ B cell lymphomas, differentiation of non-GCB DLBCL from BL and FL (j)
**CD44**	ECMRII, H-CAM, Pgp-1, Phagocytic glycoprotein I, Extracellular matrix receptor III, GP90 Lymphocyte homing/adhesion receptor, Hyaluronate receptor	Hematopoietic and non-hematopoietic cells, except platelets, hepatocytes, testis	binds hyaluronic acid, adhesion	Negative marker in FL (k) and BL (l)

**CD49d**	VLA-4	T cells, B cells, NK, thymocytes, monocytes, eosinophils, mast cells, Dendritic cells	integrin alpha4, adhesion, cell migration, homing and activation. CD49d/CD29 binds fibronectin, VCAM-1 & MAdCAM-1	Prognostic marker in CLL (m)
**CD72**	Ly-19.2, Ly-32.2, Lyb2	B-cells (but not plasma B-cells), macrophages, follicular dendritic cells, epithelial cells and endothelial cells	CD5, CD100 receptor, B-cell activation and proliferation	Negative marker for SMZL (n)
**CD74**	LN2, DHLAG, HLADG, Ia-g, li, invariant chain	B-cells, activated T-cells, macrophages, Langerhans cells, dendritic cells, endothelial cells and epithelial cells	MHC class II traffic and function, binds MIF, maturation of follicular B cells	Potential target for immunotherapy (o)
**CD79b**	IGB (Immunoglobulin-associated b), B29	B cells	Subunit of B-cell antigen receptor (CD79a+CD79b).Signal transduction.	Marker of mature B cells, Matutes scoring system (g)
**CD81**	TAPA1, S5.7, Tetraspanin-28	T cells, B cells, NK cells, thymocytes, Dendritic cells , endothelial cells, fibroblasts, neuroblastomas, melanomas	Signal transduction. Facilitates complement recognition. Complex with CD19 and CD21, signaling, T cell co-stimulation	Minimal residual disease marker in CLL (p)
**CD103**	HML-1, Integrin aE, ITGAE, OX62, HML1	Intraepithelial cells, lymph subsets, activated lymphocytes, Treg cells	Complex with integrin β7, binds E-cadherin, lymph homing/retention	Marker for HCL
**CD138**	Syndecan-1, Heparan sulfate proteoglycan	Plasma cells, pre-B-cells, epithelial cells, neural cells and breast cancer cells	Adhesion, cell morphology	Plasma cell marker
**CD183**	CXCR3, GPR9, CKR-L2, CMKAR3, IP10, Mig-R, TAC	T-cell subsets, B-cells, natural killer cells, monocytes, macrophages and proliferating endothelial cells, eosinophils, GM-CSF–activated	T-cell chemotaxis, integrin activation, cytoskeletal changes and chemotactic migration in inflammation-associated effector T-cells	Marker for CLL and MZL (q)

		CD34+ progenitors		
**CD196**	CCR6, BN-1, DCR2, DRY6, CKRL3, GPR29, CKR-L3, CMKBR6, GPRCY4, STRL22, CC-CKR-6	T cell subsets, B cells, Dendritic cell subset	binds MIP-3alpha/LARC, affects dendritic cell chemotaxis	Differential expression in B cell lymphomas (r)
**CD197**	CCR7 (formerly CDw197), BLR2, EBI1, CMKBR7	Activated T- and B-cells. Strongly upregulated in B-cells infected with Epstein-Barr	Receptor for MIP-3-beta. Mediator of Epstein-Barr virus effects on B-cells and lymphocyte migration into lymph nodes	Differential expression in B cell lymphomas (r)
**CD200**	OX2, MRC, MOX1, MOX2	Thymocytes, endothelial cells, B cells, T activated cells	Down-regulatory signal for myeloid cell function, costimulates T-cell proliferation	CLL/SLL marker
**CD220**	Insulin receptor (INSR), IR	Widely expressed in tissue targets of insulin metabolic effects	Tyrosine-protein kinase activity. Binding of insulin stimulates its association with downstream mediators including insulin receptor substrates and phosphatidylinositol 3'-kinase (PI3K), which leads to glucose uptake	Correlate with del11q- in CLL/SLL (s)
**CD305**	LAIR1	T- and B-cells, natural killer cells, dendritic cells, monocytes and macrophages	Inhibitory receptor on NK and T cells	Marker for HCLv (t)
**FMC7**	conformational epitope on the CD20	B cells	B-cell activation and proliferation	Differential expression in B cell lymphomas, Matutes scoring system (g)
**Heavy Chains (IgG, IgA, IgM,**		B cells, plasma cells	Antigen recognition	Differential expression in B cell lymphomas

**IgD)**				
**Bcl-2**	B-cell lymphoma 2	Broad	Acts promoting cellular survival and inhibiting the actions of pro-apoptotic proteins	Positive in most B cell lymphomas, overexpressed in FL, negative in BL
**MIB1**	Ki-67	cell cycle associated nuclear protein	associated with ribosomal RNA transcription	Proliferation marker

Abbreviation.
MALT: mucosa associated lymphoid tissue. CLL/SLL: chronic lymphocytic leukemia/small lymphocytic lymphoma. MCL: mantle cell lymphoma. FL: follicular lymphoma. GCB-DLBCL germinal center like diffuse large B cell lymphoma. HCL: hairy cell leukemia. SMZL: splenic marginal zone lymphoma

Table references

a. Jones RA, Master PS, Child JA, Roberts BE, Scott CS. Diagnostic differentiation of chronic B-cell malignancies using monoclonal antibody L161 (CD1c). *Br J Haematol*. 1989;71(1):43-46. doi:10.1111/j.1365-2141.1989.tb06272.x
b. Huynh MQ, Wacker HH, Wündisch T, et al. Expression profiling reveals specific gene expression signatures in gastric MALT lymphomas. *Leuk Lymphoma*. 2008;49(5):974-983. doi:10.1080/10428190802007734
c. Yoon SO, Zhang X, Freedman AS, et al. Down-regulation of CD9 expression and its correlation to tumor progression in B lymphomas. *Am J Pathol*. 2010;177(1):377-386. doi:10.2353/ajpath.2010.100048
d. Kost CB, Holden JT, Mann KP. Marginal zone B-cell lymphoma: a retrospective immunophenotypic analysis. *Cytometry B Clin Cytom*. 2008;74(5):282-286. doi:10.1002/cyto.b.20426
e. Oliver AM, Martin F, Gartland GL, Carter RH, Kearney JF. Marginal zone B cells exhibit unique activation, proliferative and immunoglobulin secretory responses. *Eur J Immunol*. 1997;27(9):2366-2374. doi:10.1002/eji.1830270935
f. Otsuka M, Yakushijin Y, Hamada M, Hato T, Yasukawa M, Fujita S. Role of CD21 antigen in diffuse large B-cell lymphoma and its clinical significance. *Br J Haematol*. 2004;127(4):416-424. doi:10.1111/j.1365-2141.2004.05226.x
g. Moreau EJ, Matutes E, A'Hern RP, et al. Improvement of the chronic lymphocytic leukemia scoring system with the monoclonal antibody SN8 (CD79b). *Am J Clin Pathol*. 1997;108(4):378-382. doi:10.1093/ajcp/108.4.378
h. Stacchini A, Chiarle R, Antinoro V, Demurtas A, Novero D, Palestro G. Expression of the CD31 antigen in normal B-cells and non Hodgkin's lymphomas. *J Biol Regul Homeost Agents*. 2003;17(4):308-315.
i. Damle RN, Wasil T, Fais F, et al. Ig V gene mutation status and CD38 expression as novel prognostic indicators in chronic lymphocytic leukemia. *Blood*. 1999;94(6):1840-1847.
j. Cardoso CC, Auat M, Santos-Pirath IM, et al. The importance of CD39, CD43, CD81, and CD95 expression for differentiating B cell lymphoma by flow cytometry. *Cytometry B Clin Cytom*. 2018;94(3):451-458. doi:10.1002/cyto.b.21533
k. Detry G, Drénou B, Ferrant A, et al. Tracking the follicular lymphoma cells in flow cytometry: characterisation of a new useful antibody combination. *Eur J Haematol*. 2004;73(5):325-331. doi:10.1111/j.1600-0609.2004.00313.x

l. Schniederjan SD, Li S, Saxe DF, et al. A novel flow cytometric antibody panel for distinguishing Burkitt lymphoma from CD10+ diffuse large B-cell lymphoma. *Am J Clin Pathol*. 2010;133(5):718-726. doi:10.1309/AJCP0XQDGKFR0HTW
m. Dal Bo M, Tissino E, Benedetti D, et al. Functional and Clinical Significance of the Integrin Alpha Chain CD49d Expression in Chronic Lymphocytic Leukemia. *Curr Cancer Drug Targets*. 2016;16(8):659-668. doi:10.2174/1568009616666160809102219
n. Hammer RD, Glick AD, Greer JP, Collins RD, Cousar JB. Splenic marginal zone lymphoma. A distinct B-cell neoplasm. *Am J Surg Pathol*. 1996;20(5):613-626. doi:10.1097/00000478-199605000-00008
o. Stein R, Mattes MJ, Cardillo TM, et al. CD74: a new candidate target for the immunotherapy of B-cell neoplasms. *Clin Cancer Res*. 2007;13(18 Pt 2):5556s-5563s. doi:10.1158/1078-0432.CCR-07-1167
p. Rawstron AC, Villamor N, Ritgen M, et al. International standardized approach for flow cytometric residual disease monitoring in chronic lymphocytic leukaemia. *Leukemia*. 2007;21(5):956-964. doi:10.1038/sj.leu.2404584
q. Jones D, Benjamin RJ, Shahsafaei A, Dorfman DM. The chemokine receptor CXCR3 is expressed in a subset of B-cell lymphomas and is a marker of B-cell chronic lymphocytic leukemia. *Blood*. 2000;95(2):627-632.
r. Wong S, Fulcher D. Chemokine receptor expression in B-cell lymphoproliferative disorders. *Leuk Lymphoma*. 2004;45(12):2491-2496. doi:10.1080/10428190410001723449
s. Saiya-Cork K, Collins R, Parkin B, et al. A pathobiological role of the insulin receptor in chronic lymphocytic leukemia. *Clin Cancer Res*. 2011;17(9):2679-2692. doi:10.1158/1078-0432.CCR-10-2058
t. Julhakyan HL, Al-Radi LS, Moiseeva TN, et al. A Single-center Experience in Splenic Diffuse Red Pulp Lymphoma Diagnosis. *Clin Lymphoma Myeloma Leuk*. 2016;16 Suppl:S166-S169. doi:10.1016/j.clml.2016.03.011
